# Unveiling the carbon-reduction potential of data sharing: A quasi-experimental investigation of public data open platforms in Chinese cities

**DOI:** 10.1371/journal.pone.0324036

**Published:** 2025-05-28

**Authors:** Chengxing Xie, Yue Wang, Weilong Wang, Liang Wu

**Affiliations:** 1 School of Economics, Sichuan University, Chengdu, China; 2 School of Economics and Management, Tarim University, Alar, China; 3 Bay Area International Business School, Beijing Normal University, Zhuhai, China; Wenzhou University, CHINA

## Abstract

Existing research predominantly delves into the environmental governance performance of the digital economy, ignoring the latent values of data factors. Public Data Open Platforms (PDOPs) have released vast amounts of public data, fostering high-level applications of data across multiple industries and domains. By employing panel data from 281 cities in China spanning 2006–2021, we empirically verify the impact of PDOPs on city-level carbon reduction, supported by various robustness tests, including the PSM-DID and placebo tests. Moreover, we emphasize that promoting green technology innovation and optimizing resource allocation efficiency are pivotal pathways through which PDOPs can enhance their carbon reduction effects. Heterogeneity analysis reveals that cities with more advanced digital infrastructure and higher market integration experience a more pronounced carbon reduction impact from PDOPs. Additionally, resource-abundant cities benefit more from PDOPs’ carbon reduction effects than resource-scarce ones.

## 1. Introduction

Addressing the climate crisis has become a global priority for achieving sustainable development [1,2]. According to the International Energy Agency’s (IEA) ‘*CO2 Emissions in 2022*’ report, China’s energy-related carbon emissions reached approximately 12.1 billion tons between 2021 and 2022, positioning the country as one of the largest carbon emitters globally [3]. Consequently, China faces a pressing and complex challenge in reducing its carbon emissions. In response, the Chinese government has introduced a range of measures aimed at mitigating carbon pollution and advancing its climate goals [4]. For instance, the 14th Five-Year Plan sets specific targets, including a 13.5% reduction in energy consumption per unit of GDP and an 18% reduction in CO2 emissions [5]. Additionally, the 2021 Central Economic Work Conference underscored the importance of establishing incentive and constraint mechanisms to support pollution and carbon emission reduction efforts [6]. Achieving the “dual carbon” goals—namely, carbon peaking by 2030 and carbon neutrality by 2060—requires a focus on controlling total carbon emissions, with an emphasis on enhancing carbon productivity. Therefore, exploring effective strategies to reduce urban carbon emissions is critical for facilitating this transition and informing the adjustment of carbon reduction policies.

To enhance data quality and maximize the value-creation potential of data factors, China has progressively introduced Public Data Open Platforms (PDOPs) at the local government level. As of August 2023, 202 cities in China had implemented PDOPs, offering a wealth of reliable and comprehensive public data resources. These platforms encompass essential data categories such as urban geoinformation, meteorological services, public health, and transportation, as well as data spanning technological innovation, social security, credit services, market regulation, resource energy, and ecological environment. In this study, we investigated the impact of data sharing on carbon emissions reduction using Chinese urban PDOPs as a quasi-natural experiment. The potential of data sharing in mitigating carbon emissions has emerged as a compelling topic in the digital age. Despite its growing recognition, the role of data as a foundational resource within the digital economy for reducing carbon emissions remains relatively unexplored. Exploring the intersection of data sharing and carbon reduction strategies could unlock new possibilities for sustainable environmental outcomes in the evolving digital landscape [7].

Data, as a virtual production factor, is distinct from traditional factors like capital and labor [8]. The characteristics of data, including cross-temporal and spatial information dissemination, sharing, and recreation [9], play a pivotal role in supporting the achievement of the “dual carbon” goals. Data provides real-time and precise insights into energy usage, offering government and enterprise decision-making assistance. This capability facilitates optimized resource allocation and boosts energy efficiency. Moreover, data-driven technological innovations drive progress in energy structures by promoting the adoption of cleaner and renewable energy sources, thereby reducing reliance on fossil fuels. The deep integration of data into carbon-intensive industries such as electricity, energy, industry, and transportation fosters the application of digital technologies and digital transformation in these sectors. This integration helps in lowering energy consumption throughout product lifecycles [10].

In our study, we analyze panel data from 281 cities spanning 2006–2021 to explore the effects of data sharing on carbon emissions reduction. Our findings highlight the remarkable effectiveness of PDOPs in lowering urban carbon intensity and per capita emissions. A series of robustness checks provide strong support for this key finding. Notably, PDOPs are an important but not the only factor promoting urban carbon reduction. Other factors such as urbanization, energy consumption structure, environmental regulation, government intervention, and financial development also play a key role in carbon reduction. Moreover, our heterogeneity analysis reveals that the carbon reduction impact of PDOPs is more pronounced in cities with advanced digital infrastructure, enhanced market integration, and those classified as resource-based. Furthermore, we uncover that fostering and promoting green technology innovation (GTI) and enhancing optimizing resource allocation efficiency (RAE) constitute effective pathways for PDOPs to facilitate carbon reduction.

The paper’s contributions are threefold: Firstly, this study enhances the theoretical and empirical understanding of “digital carbon reduction” by examining the role of data sharing, the cornerstone of the digital economy. However, existing studies focus on superficial aspects of the carbon reduction of the digital economy. For example, Shen et al. [11], Han and Jiang [12], Dong et al. [13] showed the impact of the digital economy on carbon emissions, carbon emission intensity, and carbon emission efficiency; Zeng and Yang [14] investigated how digital technology positively affects carbon emission reduction through industrial structure, technological innovation, and tax structure. Our study offers a fresh viewpoint for research at the intersection of the digital economy and carbon neutrality. Secondly, we expand on two micro-mechanisms of how data sharing impacts per capita carbon emissions and carbon intensity. By analyzing GTI and RAE, we demonstrate how public data openness strategies, guided by governmental governance and market benefits, lead to a decrease in CO2 intensity and total emissions. This insight extends the research of Wen et al. [6] and Wu & Xie [15], and aids cities in transitioning from energy consumption “dual control” to carbon “dual control.” Thirdly, we further explore the policy implications of PDOPs amidst variations in urban digital infrastructure, market integration, and resource endowments. That offers vital policy insights for refining institutional designs and unleashing the carbon reduction value of public data openness.

Subsequent sections of this paper follow: **Section 2** reviews the literature relevant to this study. **Section 3** delineates our theoretical framework and presents the research hypotheses. **Section 4** designs our econometric model, variable definitions and data sources. **Section 5** reports the results of benchmark regressions, robustness tests and heterogeneity analysis. **Section 6** details the outcomes of the mechanism tests. **Section 7** summarizes the main findings and limitations of this study.

## 2. Literature review

Existing research offers a wealth of insights into effective carbon emission reduction strategies. From an economic and social development perspective, the Environmental Kuznets Curve proposes a theoretical hypothesis that there is an inverted U-shaped relationship between CO2 emissions and income levels, suggesting that CO2 emissions will experience an upward and downward trend as future incomes increase [16]. However, the emergence of this inflection point depends on economic development reaching a certain threshold, and CO2 emissions may only gradually decrease once the economic level exceeds a specific stage. Yang et al. [17] found that per capita GDP growth and population expansion hinder the decoupling process between carbon emissions and economic growth. Feng et al. [18] demonstrated that optimizing industrial structure can reduce China’s carbon emissions, with financial development playing a pivotal moderating role. Wang et al. [19] noted that urbanization escalates energy consumption, subsequently leading to a surge in urban carbon emissions. Regarding carbon emission mitigation at the source, boosting renewable energy, optimizing energy consumption structures, and strengthening environmental regulation can effectively reduce carbon emissions [20]. Furthermore, local governments can effectively reduce regional carbon emissions by planning budgets, enhancing foreign trade, fostering technological innovation, and encouraging low-carbon consumer behavior [11]. Concurrently, as digital technology integrates further with the real economy, scholars have observed the immense potential of new models, technologies, and business formats, including digital technologies, digital industries, and digital transformation, in promoting carbon reduction [14,21].

PDOPs enhance the accessibility and inclusivity of public data, serving as a strong catalyst for businesses, industries, and regions to overcome data monopolies and bridge data silos. By facilitating public data sharing, PDOPs greatly aid enterprises in precise production, management, marketing, and product development [22], thereby enhancing energy efficiency and reducing pollution emissions. Firstly, from the enterprise production aspect, PDOPs provide real-time, multi-dimensional data to help enterprises optimize their production processes and reduce resource wastage and pollution emissions. For example, enterprises use the meteorological and soil data provided by PDOPs to monitor the humidity and fertility of farmland, precisely control irrigation and fertilizer application, reduce the waste of water and fertilizer, and reduce agricultural pollution [15]. Meanwhile, PDOPs help enterprises optimize product design and development and reduce carbon emissions during the product life cycle [23]. Specifically, enterprises obtain and analyze environmental data from PDOPs to develop green technologies and products to enhance market competitiveness. They also simulate the carbon emissions of products in different life cycles through digital technology to make low-carbon design plans and reduce product carbon emissions. Secondly, from the management aspect, PDOPs help enterprises improve their management efficiency [24]. PDOPs provide data for enterprise operations, such as market trends, supply chain information, consumer behaviors, etc. By analyzing the data, enterprises can optimize supply chain management, reduce energy consumption, develop precise marketing strategies, and improve environmental performance. Furthermore, PDOPs break down industrial and regional barriers to information flow through data sharing, reducing the knowledge threshold for global technology integration [25].

Currently, research on PDOPs predominantly concentrates on the realm of public administration. In terms of government governance, PDOPs enhance government transparency, optimize the efficiency of government services, and bolster public decision-making behaviors alongside innovative models of government-public coordination [26,27]. Within the commercial sphere, PDOPs not only reduce the cost of data acquisition for enterprises but also facilitate information mining using public data, enabling businesses to accurately grasp commercial opportunities, thereby positively impacting operational efficiency, innovation, performance, and value creation [28]. Furthermore, the institutional basis and determinants of PDOPs have been extensively discussed by several scholars [24,29].

Unfortunately, existing literature lacks direct investigation of the impact of PDOPs on urban carbon emissions. Notably, Jetzek et al. [24] argued that public data has been widely used in scenarios like enterprise green finance and urban transportation, which can generate significant green benefits through information sharing and market mechanisms. Wu & Xie [15] demonstrated that the Chinese government’s open sharing of public data has significant ecological value, which significantly reduces CO2 and triple-waste emissions from enterprises. Therefore, it is foreseeable that PDOPs may have the potential to reduce urban carbon emissions.

Additionally, data sharing has been a key topic of academic focus. Scholars have launched extensive discussions on data sharing in the fields of natural science, medicine, psychology, ecology, public health, and business. Kowalczyk & Shankar [30] argued that data sharing cuts across many disciplines, specializations and literatures. And, his study comprehensively explored issues such as policy and technology of data sharing in the natural sciences. Fan et al. [31] proposed to build electronic medical records with high security and privacy through blockchain technology, thereby achieving efficient and secure medical data sharing. Gu [32] suggested that business models such as shared knowledge education, shared healthcare, and shared food can serve to curb city carbon emissions. Moreover, Michener [33] and Martone et al. [34] also supported data sharing in the fields of ecology, public health, and psychology. However, achieving data sharing faces various challenges in ethics, security, privacy, business, and other aspects [35,36]. This also severely restricts the scale of data sharing and its potential to empower social and economic development. PDOPs are the successful practice of public data sharing, which plays an important role in city governance and green development [37].

Practically, data sharing has a major implication for carbon reduction. First, blockchain technology, 5G technology, and other technologies are used to transmit, store, and analyze the carbon data monitored in real-time, achieving tamper-proof and secure sharing of data, and ensuring the comprehensiveness, timeliness, authenticity, and reliability of carbon data [38]. Second, data sharing contributes to the optimal allocation and efficient use of resources. For example, in public transportation and logistic transport, sharing traffic demand and capacity data can help optimize the allocation of transport resources and reduce idling, congestion, and duplication of transport, thereby reducing carbon emissions [15]. It can positively affect enterprises’ production plans, delivery times, transport arrangements, etc. Specifically, enterprises use these shared data for analysis to coordinate resources better and optimize transport and production arrangements, thereby reducing unnecessary transport consumption and carbon emissions. Moreover, data sharing can break down the information barriers between various links in the industry chain and promote information sharing and collaborative decision-making [39]. This facilitates the joint promotion of carbon reduction targets. In addition, data sharing can provide policymakers with accurate carbon data to support the formulation of scientifically rational emission reduction policies [15].

## 3. Research hypotheses

### 3.1. Data sharing and carbon emission reduction

PDOPs facilitate online searches and provide access to public data resources, enabling various entities to utilize these resources effectively [40]. This significantly fosters data resource sharing. Through this mechanism, enterprises can integrate data resources into their production and management processes at minimal or no cost. This integration helps them develop optimal production and sales strategies, leading to a mutually beneficial outcome that merges resource and energy conservation with enhanced corporate performance. Consequently, this approach contributes to reducing carbon emissions [20].

Furthermore, PDOPs help alleviate information asymmetry between enterprises and investors, directing capital towards cleaner production sectors. This not only eases financing constraints for environmentally friendly enterprises but also constrains the production scales of energy-intensive and high-emission enterprises. As a result, industrial structures are reshaped towards cleaner and smarter development, ultimately leading to a reduction in urban CO2 emissions scales [41].

Moreover, the public data resources made available by PDOPs foster the integration of data across various sectors, such as public health, traffic management, ecological environment, and grassroots governance. This integration enhances urban services, management practices, and operational efficiency, leading to reduced energy and resource consumption and consequently lowering urban CO2 emissions [42].

Additionally, PDOPs provide channels for public access to data. Through this process, information concerning the ecological environment and energy resources motivates public environmental awareness, thus bolstering public engagement in environmental issues. This increased awareness puts pressure on governments to strengthen environmental regulations and encourages enterprises to proactively embrace environmental protection responsibilities, fostering the advancement of urban green low-carbon development [12].

Consequently, we propose the following hypothesis:

**Hypothesis 1:** PDOPs promote carbon reduction.

### 3.2. Impact mechanisms

#### 3.2.1 .Promoting green technology innovation.

Green technology innovation stands as a powerful strategy for curbing carbon emissions. Firstly, green innovation plays a pivotal role in providing technological support for advancing research, development, and implementation of carbon capture, utilization, and sequestration technologies. Secondly, innovations in renewable energy can enhance the utilization of cleaner energy sources, thereby facilitating the transition towards sustainable energy practices [43], directly leading to a reduction in CO2 emissions. Moreover, GTI boosts clean production efficiency and promotes the availability of green products, thereby lowering carbon emissions both in production processes and consumption patterns [44].

PDOPs significantly contribute to enhancing the level of GTI. Firstly, their transparency and real-time accessibility streamline data collaboration and resource allocation, fostering the convergence of data value chain nodes across research, development, production, and supply chains. This dismantles barriers related to information asymmetry, enabling a more effective pooling of innovative insights and capabilities. Secondly, rooted in dynamic capabilities theory, the paradigm shift fueled by data resources bolsters firms’ abilities to identify and capitalize on innovation opportunities, driving a surge in green innovation levels to better align with evolving market dynamics. Specifically, PDOPs provide real-time data in various areas, including environment, energy, market, and consumer behavior. Enterprises analyze this data to quickly sense market changes and better understand market demand, technology trends, and consumer preferences, thereby identifying potential innovation opportunities. For instance, data boosts firms’ responsiveness to green consumer preferences, guiding them towards environmentally friendly product innovations. Such a data-driven innovation model has a wide range of applications in areas like clean energy technology development, green product design, and supply chain optimization [45]. This suggests that dynamic capabilities like environmental sensing and opportunity exploitation, built on data resources, empower firms to break free from entrenched patterns in green innovation, optimizing resources through strategic upgrades and gaining an edge in emerging technological and eco-friendly domains. Thirdly, in alignment with information processing theory, the scalability and accessibility of data sharing enable firms to pinpoint their resource requirements, fostering communication and collaboration with other entities, and enabling swift access to valuable innovation-related information at minimal costs. For green innovation, PDOPs not only trim firms’ resource acquisition expenses for implementing green practices but also enhance their information processing capabilities, mitigating governance risks linked to information disparities and thereby bolstering their resolve to strategically enhance green innovation.

Therefore, we propose hypothesis 2:

**Hypothesis 2:** PDOPs reduce carbon emissions by promoting GTI.

#### 3.2.2. Enhancing resource allocation efficiency.

Improving resource allocation efficiency stands as a crucial pathway for curbing carbon emissions. Improving RAE can help reduce carbon emissions by changing how industries operate. In China, there are issues with using resources efficiently, especially in industries that consume a lot of energy but add little value. This fosters the accumulation of low-end capacity and exacerbates carbon emissions. Better RAE helps cut emissions by using energy more efficiently in economic activities. On the other hand, improved RAE promotes a balanced spatial distribution of factor supply and demand, easing interregional factor price disparities and trade barriers, thereby enhancing the nation’s overall energy efficiency and ultimately reducing carbon emissions.

PDOPs are vital for improving RAE. Firstly, sharing data resources helps spread knowledge and technology among businesses, enabling the use of digital tools to enhance production processes and energy efficiency. Integrating PDOPs with cloud computing and data mining allows companies to effectively use public data for predicting market demands and optimizing production strategies, continuously improving RAE. Secondly, the data provided by PDOPs offers valuable information for decision-makers, helping them make informed choices about capital and resource allocation, reducing resource misallocation. Digital tools enable companies to navigate large datasets efficiently, improving the allocation of resources. Thirdly, PDOPs enhance market transparency by sharing data across industries, creating a fairer business environment. Efficient companies can leverage PDOPs to attract quality resources and benefit from data-driven efficiencies, enhancing their RAE. Therefore, we propose hypothesis 3:

**Hypothesis 3:** PDOPs reduce carbon emissions by enhancing RAE.

**Fig 1:**
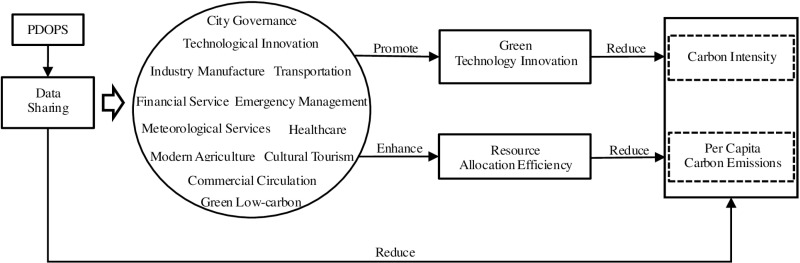
Logical framework of data sharing’s carbon-reduction effect.

Finally, according to the above theoretical analysis and research hypotheses, we summarize the logical framework diagram of this study, as depicted in Fig 1.

## 4. Method and data

### 4.1. Model design

First, drawing on the ideas of Zeng et al. [21] and Zhang et al. [46], this study adopts the carbon intensity and per capita carbon emissions of the city to test the carbon reduction effect. Second, given the data openness and sharing mechanism of PDOPs, we draw on the studies of Ma et al. [40] and Wu & Xie [15] to measure the data sharing of cities based on the establishment of PDOPs in Chinese cities. Therefore, we quantify the carbon reduction effect of data sharing based on the quasi-natural experiment of PDOPs policy by using the multi-period difference model (DID) method, which is a popular approach in policy evaluation. The DID model can capture the net effect of the establishment of PDOPs by comparing the differences between the experimental and control groups before and after the establishment of PDOPs. The multi-period difference model is as follows:


$${CI}_{it}/{CP}_{it}=\alpha_{0}+\alpha_{1}{DATA}_{it}+\alpha_{2}{Control}_{it}+\mu_{i}+\gamma_{t}+\delta_{it}$$ (1)

In Eq. (1), *CI*_*it*_ and *CP*_*it*_ serve as the dependent variables, representing the carbon emission intensity and per capita carbon emissions of the city *i* in period *t*, respectively. *CI* is measured as the ratio of urban CO2 emissions to local GDP; *CP* is represented as the ratio of urban CO2 emissions to the registered population. The urban carbon emissions data are computed based on the methodology detailed in Zhang et al. [46]. Specifically, we collected data on urban direct energy consumption (crude coal, crude oil, natural gas, etc.) and indirect electricity and heat consumption and calculated their product with the carbon emission factor, thereby obtaining urban carbon emissions. *DATA*_*it*_ is the independent variable in this study, which is a dummy variable. It takes the value of 1 if PDOPs are established in city *i* during period *t*, and 0 otherwise. Additionally, individual and time-fixed effects are denoted by *μ*_*i*_ and *γ*_*t*_*,* respectively, while *δ*_*it*_ represents the random disturbance term in the model.

To mitigate potential biases in our estimations due to omitted variables, we include a series of other factors that may impact urban carbon emissions, referred to as *Control*_*it*_. Following the literature experience of Gu [32], Wu & Xie [15], and Zhang et al. [46], our control variables include the level of economic development (*PGDP*), which represents the logarithm of per capita GDP along with its quadratic term. Additionally, we consider industrial structure (*IS*), which is characterized by the ratio of value added by the tertiary industry to GDP. Urbanization level (*Urban*) is defined as the proportion of the urban population relative to the total population. Energy consumption structure (*ECS*) is described by the percentage of electricity consumed by urban households in the overall energy consumption mix. The financial development level (*Finance*) is determined by the total deposits and loans by financial institutions in relation to annual GDP. The government intervention level (*GOV*) measures government expenditure as a portion of total economic output. Environmental regulatory intensity (*ER*) reflects the government’s importance to environmental protection and the intensity of policy enforcement. Drawing on the approach of Chen et al. [47], we measured *ER* by calculating the frequency of environmental protection-related terms in government reports using textual analysis methods. Foreign direct investment level (*FDI*) is quantified as the ratio of realized foreign investment utilization to GDP.

### 4.2. Data sources and description

The data concerning carbon emissions and the control variables utilized in our study are gathered from the City, Energy, and Industrial Statistical Yearbook of China, in conjunction with information derived from local government work reports and statistical annual reports. To ascertain the availability of urban public data platforms, we adopt the methodology outlined by Ma et al. [40] for data compilation and structuring, cross-referenced and validated with details from local government work reports, official websites, and news coverage. To guarantee data accessibility and comprehensiveness, cities with significant data gaps were omitted from the analysis, and any individual missing data points were filled in using linear interpolation. The final dataset encompasses information from 281 Chinese cities spanning the years 2006–2021. Statistical characteristics of the variables are summarized in Table 1. Wherein, PDOPs were first established in Beijing and Shanghai in 2012. Furthermore, during the study period, we collected data from a total of 196 cities with PDOPs and 85 cities without PDOPs.

**Table 1:** Descriptive statistics results.

Variables	Obs	Mean	Sd	Min	Max
CI	4496	3.608	3.806	0.131	32.994
CP	4496	0.864	0.930	0.013	3.496
DATA	4496	0.147	0.354	0.000	1.000
PGDP	4496	10.494	0.724	4.595	13.056
IS	4496	0.406	0.101	0.038	0.839
Urban	4496	0.528	0.167	0.115	1.000
ECS	4496	0.814	0.147	0.040	0.997
Finance	4496	2.325	1.165	0.560	21.302
GOV	4496	0.184	0.101	0.043	1.485
ER	4496	0.327	0.143	0.018	1.239
FDI	4496	0.018	0.020	0.000	0.229

## 5. Empirical results and discussions

### 5.1. Parallel trend test

Ensuring the assumption of parallel trends is met is a fundamental requirement for the proper utilization of the DID model. Specifically, the parallel trends assumption dictates that prior to the introduction of PDOPs, the trends in carbon emission intensity and per capita carbon emissions between pilot and non-pilot cities should follow a similar trajectory. To evaluate this assumption, we conduct a parallel trends test using the event study methodology proposed by Beck et al. [48] with the following regression model:


$$\text{C}{\text{I}}_{\text{it}}\text{/C}{\text{P}}_{\text{it}}\text{ =}\alpha_{0}\text{ + }\sum_{k=-6}^{5}\beta_{\text{k}}\text{×TREA}{\text{T}}_{\text{i,t+k}}\text{ +}{\;\alpha }_{\text{1}}\text{Contro}{\text{l}}_{\text{it}}\text{ + }\mu_{\text{i}}\text{ +}{\;\gamma }_{\text{t}}\text{ + }\delta_{\text{i,t}}$$ (2)

In Eq. (2), *TREAT*_*i,t+k*_ represents the policy dummy variable for year *t + k*. When *k* is negative, it indicates *k* years before policy implementation; when *k* is positive, it indicates *k* years after policy implementation. *β*_*k*_ denotes the regression coefficient, capturing the difference in carbon intensity and per capita emissions before and after the establishment of PDOPs. This study adopts a time window spanning six years before and five years after policy implementation for analysis.

**Fig 2:**
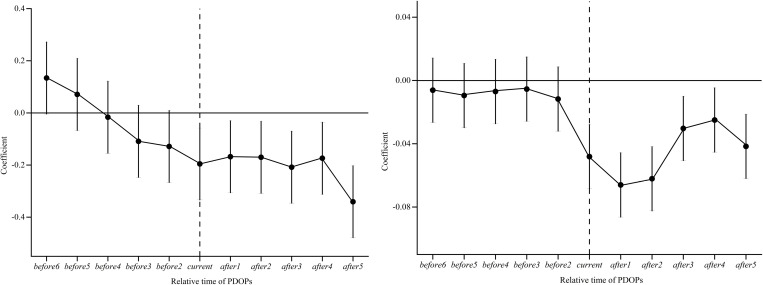
Parallel trend test. **(a)** Parallel trend test based on CI **(b)** Parallel trend test based on CP.

The results of the parallel trends test are illustrated in Fig 2. To address multicollinearity concerns, the pre-policy year immediately preceding the policy implementation is omitted from the analysis. Notably, for *k < 0*, the confidence intervals of the *β*_*k*_ estimates in both Fig 2a and 2b encompass zero. This observation indicates that prior to the introduction of PDOPs, there were no significant disparities in carbon emission intensity and per capita carbon emissions across cities, fulfilling the parallel trends test. Furthermore, when *k ≥ 0*, the β_k_ estimates are significantly negative. This implies that following the implementation of PDOPs, there has been a significant suppression in carbon emission intensity and per capita carbon emissions within pilot cities, suggesting a pronounced carbon reduction impact attributed to the PDOPs.

### 5.2. Benchmark regression results

Table 2 reports the regression results of PDOPs on CI and CP using the model outlined in Eq. (1). Columns (1) and (3) show the estimation outcomes without including control variables or fixed effects, while Columns (2) and (4) introduce city and time-fixed effects. Columns (3) and (6) further include control variables. The regression coefficients of the PDOP variable, *DATA*, across Columns (1) to (6) exhibit statistically significant negative effects at the 1% level. This suggests that PDOPs significantly reduce urban carbon intensity and per capita carbon emissions, thereby validating **Hypothesis 1**. These findings are consistent with studies by Dong et al. [13], Tang & Yang [49] and Gan et al. [50], which explored carbon emission reduction effects within the digital economy, such as in the big data industry and digital infrastructure. Unlike previous studies, our research focuses on the foundational support of digital economy development—data sharing—and confirms the suppressive effect of PDOPs on carbon emissions. This extension of existing research contributes to deepening both theoretical and empirical understanding in this area.

**Table 2:** Benchmark regression results.

Variables	CI			CP		
	(1)	(2)	(3)	(4)	(5)	(6)
DATA	-1.830^***^	-0.373^***^	-0.196^***^	-0.523^***^	-0.153^***^	-0.048^***^
	(0.093)	(0.082)	(0.070)	(0.035)	(0.014)	(0.010)
PGDP			9.309			0.388
			(5.878)			(0.447)
PGDP^2^			-0.336			-0.050^*^
			(0.273)			(0.021)
IS			-0.531			-0.075
			(0.656)			(0.055)
Urban			2.562^**^			0.410^***^
			(1.117)			(0.087)
ECS			-0.711^***^			-0.014
			(0.246)			(0.026)
Finance			0.194^**^			0.024^***^
			(0.078)			(0.009)
GOV			-4.420^**^			-0.295^**^
			(1.922)			(0.149)
ER			-0.465^***^			-0.031^*^
			(0.177)			(0.018)
FDI			2.756			0.291
			(2.447)			(0.223)
Constant	3.877^***^	3.663^***^	63.25^**^	0.941^***^	0.887^***^	0.941^***^
	(0.065)	(0.024)	(31.26)	(0.015)	(0.004)	(0.015)
City fixed	N	Y	Y	N	N	Y
Time fixed	N	Y	Y	N	N	Y
N	4496	4496	4496	4496	4496	4496
R^2^	0.029	0.876	0.901	0.040	0.967	0.983

*, **, And *** indicate significant at the 10%, 5%, and 1% levels, respectively, and values in parentheses are robust standard errors, as below.

PDOPs reduce the cost of data access and enhance the public’s willingness to participate in environmental protection. This benefits the integration of public data resources into enterprises’ production, management, and sales, unleashing the potential of public data in energy conservation and emission reduction, thereby improving energy and environmental performance. Moreover, PDOPs guide the flow of capital to high-tech and clean enterprises through the marketization of data and the accumulation of green technological tools for urban carbon reduction. Additionally, the rich data provided by PDOPs, when deeply integrated with various city departments, can significantly improve productivity and reduce energy waste, thereby reducing urban carbon emissions scale and intensity.

In interpreting the impact of control variables based on Columns 3 and 6 of the analysis. The results for the variable “Urban” are consistent with the findings of Liu & Liu [51], who argue that the ongoing urbanization in China leads to increased fossil fuel consumption, thereby heightening CO2 emissions and establishing a direct positive relationship with both urban carbon intensity and per capita carbon emissions. Contrastingly, the positive and statistically significant coefficient for “Finance” at the 5% level suggests that the development of China’s financial sector plays a crucial role in driving up urban carbon emissions. This finding diverges from the conclusions of Shahbaz et al. [52] and Ren et al. [53] but aligns with the perspectives of Acheampong [54]. He argued that financial advancement enables households and businesses to access affordable credit, thereby facilitating the acquisition of energy-intensive machinery and equipment, potentially leading to increased carbon emissions. Conversely, the negative estimates for “GOV” and “ER”, significant at least at the 10% level, suggest that local Chinese governments can effectively mitigate urban carbon emissions by increasing fiscal expenditures and enforcing stricter environmental regulations. This finding is supported by Cao et al. [55] and Wang & Zhang [56]. Moreover, the estimate for “ECS” in Column 3 reveals a negative value and it is significant at the 1% level, indicating that optimizing the coal-dominated energy consumption structure can effectively reduce urban carbon intensity, as suggested by Yu et al. [57]. The coefficients for the remaining control variables are insignificant, indicating that they do not serve as primary determinants of urban carbon emissions.

### 5.3. Robustness tests

We have undertaken the following robustness test to validate the results:

1. **Substitute the dependent variable.** We replaced the dependent variable with carbon emissions per unit of non-agricultural output to represent the urban CO_2_ scale. As shown in Column (1) of Table 3, the variable *DATA* is significant at the 5% level. This significance suggests that PDOPs continue to exhibit a robust impact on urban carbon reduction.
2. **Adjustment of study period.** Considering the impact of the COVID-19 pandemic on corporate production and operations, which led to reduced resource utilization like oil and coal, we extended the study period from 2006 to 2019. By excluding the effects of pandemic shocks on urban CO_2_ emissions, Column (2) of Table 3 demonstrates that the coefficient of *DATA* remains significant at the 5% level. This consistency reinforces the stability and reliability of the above findings.
3. **Heckman’s two-step method.** Recognizing that PDOPs may not follow random sampling principles due to selective opening by government departments, we utilized the Heckman two-step method to address sample selection bias. In the first stage, we employed the Probit model with *DATA* as the dependent variable and introduced the annual mean of *DATA* (*M_DATA*) along with relevant control variables to generate the Inverse Mills Ratio (*IMR*). In the second stage, incorporating the *IMR* into the model, the coefficients of *DATA* in Columns (3) and (4) of Table 3 remain negative at the 1% level, reinforcing the reliability of the baseline regression findings.
4. **PSM-DID.** We also adopt the PSM-DID model based on the nearest neighbor matching method to address sample selection bias. Column (5) of Table 3 demonstrates that *DATA*’s coefficient remains negative at the 1% level, reconfirming the reliability of the baseline regression findings.
5. **Exclude other policy interference.** To eliminate potential interference from other policies on urban carbon emissions, we further incorporate the low-carbon city policy (*DID1*), the carbon trading pilot policy (*DID2*), and the green finance reform and innovation pilot zone policy (*DID3*) into the model. As shown by Columns (6) to (9) of Table 3, *DATA*’s coefficients are all significantly negative, which suggests that our conclusions are not affected by these policy shocks.

**Table 3:** Robustness tests.

Variables	(1)	(2)	(3)	(4)	(5)	(6)	(7)	(8)	(9)
DATA	-0.247^**^ (0.097)	-0.149^**^ (0.094)		-0.177^***^ (0.067)	-0.153^**^ (0.066)	-0.195^***^ (0.071)	-0.171^**^ (0.077)	-0.195^***^ (0.070)	-0.170^**^ (0.0764)
M_DATA			3.867^***^ (0.154)						
IMR				-0.083 (0.060)					
DID1						-0.006 (0.100)			0.0328 (0.102)
DID2							-0.167 (0.107)		-0.184^*^ (0.105)
DID3								-0.174 (0.176)	-0.176 (0.173)
Constant	100.5^**^ (48.74)	83.86^***^ (0.314)	-32.70^***^ (12.09)	134.6^***^ (7.612)	130.2^***^ (7.729)	-0.006 (0.100)	63.60^**^ (31.48)	63.16^**^ (31.31)	63.44^**^ (31.60)
Control variables	Y	Y	Y	Y	Y	Y	Y	Y	Y
City fixed	Y	Y	Y	Y	Y	Y	Y	Y	Y
Time fixed	Y	Y	Y	Y	Y	Y	Y	Y	Y
N	4496	3934	4496	4496	4452	4496	4496	4496	4496
R^2^	0.885	0.858	0.528	0.913	0.913	0.901	0.901	0.901	0.901

For brevity, only regressions on carbon intensity are presented in Columns 1–9 of the table. Detailed results are available upon request.

**Fig 3:**
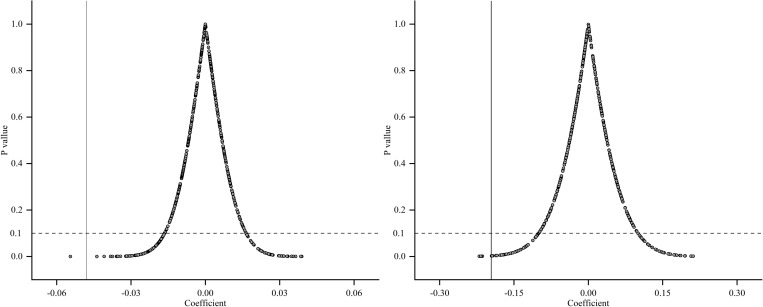
Placebo tests. **(a)** Placebo test based on CI **(b)** Placebo test based on CP.

1. **Placebo tests.** We have conducted placebo tests to assess the impact of unobservable factors on urban CO2 emissions. In these tests, pilot cities were randomly assigned, and 500 random samplings were performed. Fig 3 depicts the results, showing that the regression coefficients tend to cluster around zero. Most of the estimation coefficients did not pass the significance test. This outcome indicates that the randomly sampled city combinations did not have a significant impact on urban carbon emissions.

### 5.4. Heterogeneity analysis

The preceding sections validated the role of PDOPs in urban carbon emission reduction when considering the entire sample. In the next step of the analysis, the study will conduct a heterogeneity analysis to investigate whether PDOPs have varying impacts on urban carbon reduction outcomes based on differences in cities’ characteristics, specifically focusing on digital infrastructure (DI), market integration (MI), and resource endowments (RE).

#### 5.4.1. Digital infrastructure.

Digital infrastructure encompasses an innovative system driven by data and rooted in communication networks and computing capabilities [49]. Cities with robust telecommunications infrastructure are crucial in facilitating information dissemination, technology sharing, production acceleration, and factor mobility [58]. This, in turn, enhances the construction and operational efficiency of PDOPs, unlocking their potential for energy-saving and emission reduction. Conversely, in cities with lower levels of digital infrastructure, the construction and maintenance of DI may involve substantial energy consumption and environmental risks [52].

To test the hypothesis regarding the impact of DI on the effectiveness of PDOPs in reducing urban carbon emissions, we utilized the DI indexes calculated based on the methodology outlined by Zhang et al. [58]. The sample was then divided into two distinct groups, categorized as high and low DI, with the average DI value serving as the threshold for segmentation.

In Table 4, Columns (1) and (2) present the effects of PDOPs on the intensity of urban carbon emissions across different DI groups. Similarly, Table 5, Columns (1) and (2), illustrate the impact of PDOPs on urban per capita carbon emissions within various DI categories. Significantly, in the high-DI group, the coefficients for the variable *DATA* are significant and negative at the 1% level. In contrast, the coefficients in the low-DI group do not reach statistical significance. This outcome aligns with our expectations, indicating that a strong digital infrastructure enhances the carbon reduction effectiveness of PDOPs. Furthermore, this result supports the findings of Zhang et al. [58], who suggest that the energy rebound phenomenon linked to digital infrastructure development is temporary. Over the long term, DI emerges as a critical factor in achieving substantial carbon reduction benefits.

**Table 4:** Heterogeneity analysis based on carbon intensity.

Variables	Digital infrastructure		Market integration		Resource endowment	
	High	Low	High	Low	RBCs	Non-RBCs
	(1)	(2)	(3)	(4)	(5)	(6)
DATA	-0.211*** (0.059)	-0.064 (0.113)	-0.253** (0.101)	-0.053 (0.114)	-0.324** (0.133)	-0.052 (0.079)
Constant	108.2*** (36.79)	45.89 (32.18)	52.15* (28.52)	136.4*** (15.19)	124.9*** (9.716)	28.23 (25.82)
Control variables	Y	Y	Y	Y	Y	Y
City fixed	Y	Y	Y	Y	Y	Y
Time fixed	Y	Y	Y	Y	Y	Y
Chow test	chi2 = 17.09 Prob > chi2 = 0.000		chi2 = 5.04 Prob > chi2 = 0.025		chi2 = 11.01 Prob > chi2 = 0.001	
N	1252	3173	2885	1609	2712	1776
R^2^	0.954	0.907	0.900	0.929	0.898	0.911

**Table 5:** Heterogeneity analysis based on per capita carbon emissions.

Variables	Digital infrastructure		Market integration		Resource endowment	
	High	Low	High	Low	Abundant	Scarce
	(1)	(2)	(3)	(4)	(5)	(6)
DATA	-0.049*** (0.014)	-0.007 (0.013)	-0.056*** (0.014)	-0.031* (0.016)	-0.041*** (0.012)	-0.019 (0.015)
Constant	5.676* (3.095)	0.735 (2.470)	1.017 (2.003)	3.887*** (0.461)	5.682*** (0.833)	-0.212 (2.132)
Control variables	Y	Y	Y	Y	Y	Y
City fixed	Y	Y	Y	Y	Y	Y
Time fixed	Y	Y	Y	Y	Y	Y
Chow test	chi2 = 5.80 Prob > chi2 = 0.016		chi2 = 2.54 Prob > chi2 = 0.111		chi2 = 3.44 Prob > chi2 = 0.064	
N	1252	3173	2885	1609	2712	1776
R^2^	0.987	0.981	0.984	0.987	0.986	0.980

#### 5.4.2. Market integration.

The market integration stands as a crucial determinant of the efficacy of PDOPs in reducing urban carbon emissions. Our study delves into high- and low- market integration scenarios to explore this relationship in depth.

Higher levels of MI are associated with increased factor mobility, improved data sharing effectiveness, and enhanced RAE for data, labor, capital, and technology [59]. Technologically, MI facilitates the flow of energy-saving and emission-reduction technologies within and across regions, reducing innovation costs for enterprises and amplifying the effects of technological innovations on emission reduction [60]. Market Integration also stimulates market competition, promoting the commercialization of clean energy resources and compelling enterprises to enhance energy efficiency, thereby driving urban carbon reduction efforts [61]. Furthermore, MI accelerates the marketization of data factors, enabling their circulation and unlocking their potential for “digital decarbonization” [62].

To test this hypothesis, we leverage the methodology proposed by Zhang et al. [59] to measure MI using a relative price approach. The detailed calculations are as follows: We selected the Consumer Price Index (*P*) for 7 categories in 281 cities (*N*), including food, tobacco and alcohol, clothing, household equipment, health care, transport and communications, and entertainment, education, and culture. Then, the absolute value of the relative price of goods *k*, $\left|\text{∆}Q_{ijt}^{k}\right|$, is calculated:


$$\left|\text{∆}Q_{ijt}^{k}\right|\text{= }\left|\text{ln(}\frac{P_{it}^{k}}{P_{i,t-1}^{k}}\text{) - ln(}\frac{P_{jt}^{k}}{P_{i,j-1}^{k}}\text{)}\right|\text{ = }\left|\text{ln(}\frac{P_{it}^{k}}{P_{jt}^{k}}\text{) - ln(}\frac{P_{i,t-1}^{k}}{P_{i,j-1}^{k}}\text{)}\right|$$ (4)

Further, we need to de-mean goods prices. We calculated the mean $\overset{―}{\left|\text{∆}Q_{t}^{k}\right|}$ of the relative prices of goods k to obtain the relative price change:


$$q_{ijt}^{k}=\left|\text{∆}Q_{ijt}^{k}\right|-\overset{―}{\left|\text{∆}Q_{t}^{k}\right|}$$ (5)

Based on the value of $q_{ijt}^{k}$, we can judge the fluctuation of market prices of 7 categories of goods between city *i* and city *j* in year *t*. And, its standard deviation VAR($q_{ijt}^{k}$) reflec*t*s the degree of market segmentation, the larger VAR($q_{ijt}^{k}$) the lower the degree of market integration [59]. Lastly, we merged the price differences of the above 7 categories of goods, and thus obtained a market segmentation index at the city level as a measure of the city’s market integration ${MI}_{it}=\frac{\sum_{k=1}^{k}VAR(q_{ijt}^{k})}{N}$.

Then, the sample is divided into high-MI and low-MI groups based on the mean MI value for comparative analysis. Columns (3) and (4) in Table 4 demonstrate the impact of PDOPs on carbon emission intensity across different MI levels. Notably, in high-MI cities, the coefficient for *DATA* is negative at the 5% level, indicating a strong effect. Conversely, in low-MI cities, the coefficient for DATA lacks statistical significance. It suggests that higher levels of MI enhance the effectiveness of PDOPs in reducing urban carbon intensity.

In Table 5, Columns (3) and (4) showcase the influence of PDOPs on per capita carbon emissions across varying MI levels. The results reveal that in high-MI cities, the coefficients for *DATA* are negative at the 1% level, indicating a substantial impact. Conversely, in low-MI cities, these coefficients attain significance only at the 10% level, underscoring the favorable condition of higher MI levels for enhancing the effectiveness of PDOPs in carbon reduction efforts.

#### 5.4.3. Resource endowment.

The resource endowment of a city plays a significant role in determining how PDOPs contribute to carbon reduction efforts. Our aim is to explore the heterogeneous relationship of PDOPs in carbon reduction within resource-abundant and resource-scarce scenarios.

Resource-abundant cities, abundant in natural resources, possess comparative advantages in attracting capital and labor, facilitating capital and talent accumulation, and adopting advanced green production technologies [63]. However, these cities often heavily rely on labor and mineral resource inputs, favoring energy-intensive industries and resource extraction activities that contribute to resource depletion, increased energy usage, and a rise in carbon dioxide emissions. Through data resource sharing, PDOPs can mitigate energy consumption, optimize RAE, and minimize resource wastage. Moreover, data sharing encourages traditional energy enterprises to undergo digital transformation [64], fostering GTI and development, enhancing energy efficiency, and substantially reducing carbon emissions in resource-based cities [65]. Consequently, PDOPs can significantly reduce fossil energy consumption, improve energy efficiency in resource-abundant cities, enhance environmental governance efficiency, and lead to substantial carbon emission reductions. In contrast, for non-resource-based cities, the carbon reduction effects of PDOPs may exhibit diminishing marginal returns.

To investigate this, we divided the samples into resource-abundant and resource-scarce cities following the guidelines outlined in the ‘*National Sustainable Development Plan for Resource-Based Cities (2013-2020)*’. Specifically, we identified the resource endowment characteristics of the 281 cities in the sample and defined the growing, mature, declining, and regenerating cities as resource-abundant cities and the others as resource-scarce cities. Ultimately, we obtained 110 resource-abundant and 170 resource-scarce cities. Based on the grouped samples, we conducted tests on carbon emission intensity and per capita carbon emissions separately. Columns (5) and (6) in Table 4 depict the impact of PDOPs on urban carbon emission intensity concerning varying urban resource endowments. Likewise, Columns (5) and (6) in Table 5 illustrate their impact on urban per capita carbon emissions. It is evident that within the resource-abundant city group, the estimated coefficient of *DATA* is negative, at least at the 5% level. In contrast, in the resource-scarce city group, the *DATA* coefficients do not exhibit statistical significance. It suggests that PDOPs have the potential to significantly reduce both carbon intensity and per capita emissions in resource-abundant cities. However, the carbon reduction effect of PDOPs on resource-scarce cities is not as apparent.

## 6. Mechanism tests

### 6.1. Mechanism test model

In this paper, a two-step mechanism testing model is employed. Firstly, drawing on Wang et al. [66], the relationship between PDOPs and mechanism variables is verified using the following model:


$$\text{Me}{\text{d}}_{\text{it}}\text{ = }\alpha_{\text{0}}\text{ + }\alpha_{\text{1}}\text{DAT}{\text{A}}_{\text{it}}\text{ + }\alpha_{\text{2}}\text{Contro}{\text{l}}_{\text{it}}\text{ + }\mu_{\text{i}}\text{ + }\gamma_{\text{t}}\text{ + }\delta_{\text{it}}$$ (6)

In Eq. (6), *Med*_*it*_ represents the mechanism variables incorporating GTI and RAE, while the other variables maintain their meanings as in Eq. (1). To quantify GTI, the logarithm of the total count of city-level green invention patents and green utility model patents is utilized, following the methodology outlined by Gao et al. [67]. Furthermore, drawing on Hsieh & Klenow [68], the C-D function is employed to calculate resource misallocation in cities as an indicator of RAE, where a higher RAE signifies lower efficiency in resource allocation.

Furthermore, to enhance the reliability of this mechanism testing model, the samples are categorized according to the mean values of the mechanism variables, and grouped regressions are performed.

### 6.2. Mechanism test results

In the analysis of the mechanisms of data sharing on carbon reduction through GTI, Table 6 reveals significant insights. Column (1) highlights a statistically significant positive regression coefficient for *DATA*, indicating that data sharing fosters GTI at the 5% level. Columns (3) and (5) demonstrate that in cities with advanced GTI capabilities, *DATA*’s estimated coefficients are significantly negative at 1%. In contrast, Columns (2) and (4) suggest that the carbon reduction impact of PDOPs is inconsequential in cities with low GTI. These results imply that PDOPs can effectively reduce carbon emissions by promoting GTI, thus affirming **Hypothesis 2**.

**Table 6:** Mechanism test – GTI.

Variables	GTI	CI		CP	
		GTI≤Mean	GTI>Mean	GTI≤Mean	GTI>Mean
	(1)	(2)	(3)	(4)	(5)
DATA	0.015^**^ (0.006)	-0.063 (0.055)	-0.244^***^ (0.092)	-0.010 (0.014)	-0.080^***^ (0.013)
Constant	1.380^***^ (0.244)	32.60^*^ (19.07)	233.0^***^ (9.425)	-0.265 (1.748)	8.788^***^ (1.143)
Control variables	Y	Y	Y	Y	Y
City fixed	Y	Y	Y	Y	Y
Time fixed	Y	Y	Y	Y	Y
Chow test	——	chi2 = 11.91 Prob > chi2 = 0.001		chi2 = 11.76 Prob > chi2 = 0.001	
N	4496	2390	2087	2390	2087
R^2^	0.981	0.904	0.943	0.979	0.982

Practically, high GTI capabilities stimulate innovations in crucial areas like CCUS and renewable energy technologies, which stand out as highly effective means of carbon reduction [69]. Simultaneously, the openness, transparency, and cost-effectiveness of PDOPs dismantle barriers to information flow. This flow enhances enterprises’ awareness of green consumer preferences, aiding them in moving away from traditional innovation paths. Consequently, this allows them to seize first-mover advantages in new technological domains and environmentally friendly sectors, fostering a proactive approach towards green innovation. Ultimately, driving GTI emerges as a pathway to amplify the carbon reduction impacts of PDOPs.

Table 7 presents the test results regarding the impact of data sharing on carbon reduction through the mechanism of RAE. In Column (1), a statistically significant negative regression coefficient for DATA at the 5% level indicates that PDOPs can significantly improve RAE. Columns (2) and (4) demonstrate that in cities with higher RAE, the coefficients of *DATA* are negative at 5% the level. In contrast, Columns (3) and (5) show that the carbon reduction effects of PDOPs do not exhibit statistical significance in cities with low RAE. These results suggest that PDOPs can foster carbon reduction by enhancing RAE, thus validating **Hypothesis 3**.

**Table 7:** Mechanism test – RAE.

Variables	RAE	CI		CP	
		RAE≤Mean	RAE>Mean	RAE≤Mean	RAE>Mean
	(1)	(2)	(3)	(4)	(5)
DATA	-0.017^**^ (0.007)	-0.408^***^ (0.114)	0.051 (0.076)	-0.034^**^ (0.0138)	-0.038 (0.0267)
Constant	3.063^***^ (0.968)	45.32 (35.24)	85.77^***^ (7.858)	-0.219 (1.908)	8.446^***^ (1.101)
Control variables	Y	Y	Y	Y	Y
City fixed	Y	Y	Y	Y	Y
Time fixed	Y	Y	Y	Y	Y
Chow test	——	chi2 = 12.69 Prob > chi2 = 0.000		chi2 = 4.10 Prob > chi2 = 0.043	
N	4496	2463	2001	2463	2001
R^2^	0.711	0.908	0.939	0.983	0.988

On one hand, augmented RAE reduces carbon emissions by reshaping traditional heavy industrial frameworks. On the other hand, data sharing within PDOPs expedites the spread of knowledge and technology among enterprises, facilitating the efficient utilization of public data. This enables enterprises to anticipate market demands, understand consumer preferences, devise optimal production strategies, and make informed decisions regarding resource and capital allocation, thereby continuously refining their RAE. Consequently, augmenting RAE emerges as a pathway through which PDOPs drive carbon reduction.

## 7. Conclusions, implications and limitations

### 7.1. Conclusions

With the rapid growth of China’s digital economy, the pervasive application and deep integration of data usage across diverse industries and sectors have become prominent. Facilitating the sharing and enhanced integration of data sharing, along with their advanced application in various domains, is pivotal for bolstering green, low-carbon development. In recent years, the Chinese government has gradually launched PDOPs, opening up and sharing large-scale public data resources. This has unleashed the huge potential for utilising data as a factor of production to mitigate carbon emissions. However, existing literature has limited empirical evidence of whether and how PDOPs influence urban carbon emissions. Therefore, based on PDOPs, a quasi-natural experiment, and adopting a novel approach centered on data sharing, we explore the urban carbon reduction function of PDOPs. We reveal the main findings as follows:

(1) PDOPs have positive carbon reduction effects. Specifically, PDOPs significantly reduce carbon intensity and per capita carbon emissions in urban areas. A series of robustness tests support this finding. (2) We reveal two important mechanisms by which PDOPs achieve carbon reduction. First, PDOPs can promote GTI, and high-level GTI can reduce urban carbon intensity and per capita carbon emissions. Second, PDOPs can reduce the degree of resource mismatch and then achieve carbon reduction by optimizing RAE. (3) Heterogeneity analyses show that differences in city digital infrastructure, market integration, and resource endowment significantly affect the emission reduction effect of PDOPs. Specifically, the cities with better digital infrastructure and higher market integration, the more pronounced the effect of PDOPs in promoting carbon reduction. Additionally, PDOPs demonstrate a more substantial carbon reduction effect on resource-abundant cities compared to resource-scarce cities.

### 7.2. Policy implications

The above findings provide the following policy implications for promoting data sharing and urban carbon reduction:

Firstly, promote the extensive opening and sharing of data resources. To achieve the goal of carbon neutrality, governments should prioritize the promotion of openness and the sharing of data resources to give full play to the potential of data in carbon reduction. On the one hand, it should formulate data opening standards, clarify the scope and authority of data sharing, and ensure data security and privacy protection. On the other hand, promote cross-sectoral and cross-regional data sharing through PDOPs to break down data silos and achieve efficient data circulation and utilisation. In addition, taking advantage of China’s mega-market, massive data, and rich application scenarios, it has promoted data collection, integration, and shared use in multiple fields.

Secondly, maximize the role of data in optimizing traditional factor allocation and promoting GTI. On the one hand, PDOPs contribute to carbon reduction by enhancing RAE. Thus, government leadership is required to actively promote the opening and sharing of data resources, guide regional resource integration and factor allocation through market competition mechanisms, alleviate improper resource allocation and market distortions, and improve energy efficiency. On the other hand, PDOPs promote GTI to aid in urban carbon reduction. Consequently, establishing data-sharing mechanisms is essential for enterprises to reduce risks and costs associated with GTI. Simultaneously, directing capital flows towards energy conservation, emission reduction, and clean energy sectors and strengthening the application and promotion of green technologies are also crucial. Furthermore, introducing next-generation information technologies like cloud computing and big data to integrate data into the production system can maximize the carbon reduction effects of data sharing.

Thirdly, the supporting roles of digital infrastructure, market integration, and resource endowments should be strengthened. Initially, build an information-sharing network through network facilities, data communication, and IoT infrastructure, enhance digital talent exchange and technical training, boost digital soft power, and fully tap into the potential of data sharing for carbon reduction. Secondly, continue to deepen market integration, promote and refine cross-regional mobility mechanisms for technology, talent, and capital, facilitate the marketization of data elements, and unleash their full potential. Lastly, based on regional development disparities, implement city-specific policies, capitalize on locational and industrial advantages, formulate policies for data-industry integration, and fully exploit the economic benefits and carbon reduction effects of data sharing.

Finally, emphasizing the data-sharing mechanism of PDOPs is a vital path to achieving “digital carbon reduction.” Although the development of the digital economy inevitably brings about energy consumption and carbon emissions, this problem can be effectively mitigated through the data-sharing mechanism of PDOPs. The government should prioritize support for the construction of PDOPs and guide leading digital technology enterprises and social organizations to participate in data sharing, breaking down data silos and bridging the digital divide. Meanwhile, through the synergy between data sharing and green technologies, a win-win situation for economic development and carbon emission reduction can be achieved.

### 7.3. Limitations and future directions

Admittedly, the study faces its own limitations. Firstly, although we have pinpointed the mechanisms of GTI and RAE by which PDOPs mitigate carbon emissions, there may exist additional crucial influencing mechanisms warranting further investigation. Secondly, the cross-regional distribution of data could result in spatial spillovers originating from PDOPs. Our future research efforts will focus on examining the spatial impact of PDOPs on carbon emissions.

## Supporting information

S1 Dataset Underlying data. (XLSX)
